# Macedonia in War-Time

**Published:** 1916-12-30

**Authors:** 


### MACEDONIA IN WAR-TIME.

### By an Officer of the R.A.M.C. and the Author of "Nature Notes."

War areas have bounds. Beyond the bounds life is normal. This is fortunate for those engaged in war, for it enables them to withdraw from the boredom of the Front and the interruptions of lines of communication and to enjoy a brief space of peace. We turned to the east from that crowded road that takes supplies to the Front and rode along a ridge of hills following a narrow path. In a few minutes we were beyond the bounds. The path, rough, narrow, and steep, wound, zigzag fashion, amongst rocks and prickly scrub down one side of a deep ravine at the bottom of which a shallow torrent ran towards the valley. Nothing of the war was to be heard nor seen. A huge hawk glided along the mountain-side. Many magpies flitted amongst the bushes, small birds hunted insects in the scrub. We came suddenly on a youth tending goats. He was picturesque, in blue clothes with scarlet girdle and sandals bound to his feet by leather thongs wound up to the knee. The path became dangerous to ride. We walked, our horses climbing carefully behind us. At the bottom was a water-mill and a small company of venerable Turks with ponies packed with bags of flour. The Intelligence officer talked with them and got directions for the way. We watered our horses, waded the stream, and began the steep ascent. It was long before the track got good enough to allow of riding again, but when it did it was not long before the village to which the Intelligence officer and I were bound came in sight. Flamour is a Turkish village of Macedonia, remote, high in the hills; it is in a rough shallow basin 2,000 feet above the sea, reached by rough tracks suited only to donkeys and pack-ponies. Three hundred or so of red-tiled dwellings, each with a separate courtyard secluded by a high wall and all placed without order or definite streets upon the slopes, house the population. The filth without the village and the filth in the crooked ways between the houses is horrible. Thin, mangy dogs and hens are all there is of sanitary service. The village life centres at the mosque, the minaret of which tapers up above all the houses. As we came into the village the priest was calling the faithful to prayer. Beside the mosque, under a distorted sycamore and around it, was a small market-place, and since it was market-day this place was filled with a crowd of men in gala clothing, chiefly blue with scarlet girdles, fez, and turban. The scene was bright and picturesque. There was goat meat exposed for sale, and trays of sweetmeats and raw hides. A harness-mender and a cobbler plied their trade on the rough pavement. We met the headman, who, with some of the elders of the village, came to greet us. They saluted, and shook hands gravely and courteously. Two youths took charge of our horses. The Intelligence officer talked of many things, now and again telling me what he said and the replies when he thought the conversation might interest me. He interpreted my remarks, and so brought me into the conversation. Presently one of the chief men asked us to come to his house and drink coffee. We accepted. Our host led us to a door in a high wall, entered, and bade us welcome. The house stood before us. At the height of six or seven steps above the ground was a verandah, spacious, with doors opening from it into the inner parts of the house on two sides, open to the air on other two sides. Household and agricultural implements were in courtyard and verandah. All was clean and tidy, in striking contrast to the filth in the alleys. Our host led the way up the steps into the verandah and there stood aside, and, with a courteous gesture, invited me and my companion to enter a room of the house. I found myself in a low room, square, with a roof overhead of roughly axe-hewn beams. On a flat hearth was a bright wood fire. The hearth was carefully swept, and round it were rugs and pillows that seemed to play a double part: seats by day and beds by night. On one wall hung long strings of onions. Great jars of dark earthenware of graceful shape stood beneath a deep shelf on which were piled heaps of carefully folded rugs. The walls were of mud, here and there recessed into open cupboards in which stood small household utensils—cups, tins, and so forth. I was invited to seat myself on a rug, our host and his friends, who had all removed their shoes in the verandah—a politeness our footgear prevented us showing—completed the circle round the fire. Our host took down a small saucepan with a long handle and a shallow lip and proceeded to boil water in it, carefully piling scraps of wood against the sides with a slender pair of tons. He added coffee and sugar, cleared the mixture with a few drops of cold water, and served us in tiny cups.

We sipped coffee, talked, and smoked. Our host went out of the room and entered another. Household bustle began. A veiled woman flitted to and fro across the verandah. After one of his short absences our host came to me and hung a clean towel over my shoulder. He did the same to the Intelligence officer. The latter said, "They are going to give us a meal." I replied, "Accept; they have been so very polite." Our host brought a basin and an ewer of water. He poured water over our hands, and then brought in a large round tray, with a stand beneath it, and set it on a cloth before us. On the tray was a dish of eggs cooked in fat and flavoured with cheese and spice, a dish of small cubes of white cheese, a bowl of honey, and many slices of brown bread arranged in a circle round the rim of the tray. Beside the tray was a large bowl of curds. Neither knife and fork nor spoon was provided, excepting one wooden spoon for the curds. The Intelligence officer, equal to the occasion and experienced from former travels, took a piece of bread, and, dipping into the dish, successfully attacked the eggs. I, closely imitating him, succeeded beyond my expectation. The dish proved excellent on trial. The cheese was of pleasant, salty flavour; the bread rather sour to my taste; the curds sharply acid, but agreeable to eat, leaving a pleasant, clean taste in the mouth. I am very fond of honey, but feared to try my unskilled fingers in the bowl. When we had finished a really excellent meal, our host removed the tray, brought more water to pour over our hands, and, seating himself, continued the conversation. Many subjects were touched on. I was interested to hear that the assembly expressed a wish that Great Britain should take over the country, because then "there would be peace and all men would become prosperous." These unfortunate people have been distracted by wars, persecutions, and brigandage for many years.

When we bade good-bye to our courteous hosts we took another path, and so wound our way along a narrow and precipitous track till we struck the Struma valley and climbed to our bivouacs on the hills. The potential wealth of Macedonia is fabulous. The valleys could grow produce to supply millions of men. The hills under a forestry system would grow timber of incalculable value where now a hundred acres keep one goat.

